# Peginterferon alpha-based therapy for chronic hepatitis B focusing on HBsAg clearance or seroconversion: a meta-analysis of controlled clinical trials

**DOI:** 10.1186/1471-2334-11-165

**Published:** 2011-06-09

**Authors:** Wen-cong Li, Mao-rong Wang, Ling-bo Kong, Wei-guang Ren, Yu-guo Zhang, Yue-min Nan

**Affiliations:** 1 Department of Traditional and Western Medical Hepatology, Third Hospital of Hebei Medical University, Shijiazhuang, China; 2 Department of Infectious diseases, Nanjing 81 Hospital, Nanjing 210002, China

**Keywords:** hepatitis B, HBsAg, peginterferon, interferon, lamivudine

## Abstract

**Background:**

Interferon alpha (IFNα) therapy has been widely used in the treatment of chronic hepatitis B (CHB) for decades. Nucleos(t)ide analogues are also increasingly used to treat CHB recently. More and more studies are being carried out concerning the clearance or seroconversion of HBsAg, which is recognized as an ideal goal of CHB therapy. This study conducted a meta-analysis to estimate the effect of pegylated interferon alpha (peginterferon α, PEG-IFNα)-based therapy on HBsAg clearance or seroconversion in CHB.

**Methods:**

All available controlled clinical trials, published from 2004 to 2010, with the following antiviral therapies for CHB patients: PEG-IFNα combined with lamivudine (LAM), PEG-IFNα only, conventional IFNα and LAM, with a course ≥24 weeks, were meta-analysed for HBsAg clearance and seroconversion.

**Results:**

Fourteen trials (involving a total of 2,682 patients) were identified, including seven high-quality and seven low-quality studies. The analysis results of the different antiviral therapies on HBsAg clearance or seroconversion were as follows: 1. No significant difference in HBsAg clearance or seroconversion was observed between the combination therapy group and PEG-IFNα monotherapy group [odds ratio (OR) = 1.16, 95% confidence intervals (CI) (0.73-1.85), *P *= 0.54 and OR = 1.07, 95% CI (0.58-1.97), *P *= 0.82, respectively]; 2. HBsAg clearance and seroconversion rates in patients with combination therapy were markedly higher than in those with LAM monotherapy [OR = 9.41, 95% CI (1.18-74.94), *P *= 0.03, and OR = 12.37, 95% CI (1.60-95.44), *P *= 0.02, respectively]; 3. There was significant difference in HBsAg clearance between the PEG-IFNα group and IFNα monotherapy group [OR = 4.95, 95% CI (1.23-20.00), *P *= 0.02], but not in seroconversion [OR = 2.44, 95% CI (0.35-17.08), *P *= 0.37]; 4. PEG-IFNα was superior to LAM in HBsAg seroconversion [OR = 14.59, 95% CI (1.91-111.49), *P *= 0.01].

**Conclusions:**

PEG-IFNα facilitated HBsAg clearance or seroconversion in CHB patients. PEG-IFNα-based therapy was more effective than LAM monotherapy in achieving HBsAg clearance or seroconversion for both HBeAg-positive and HBeAg-negative CHB patients. There was no significant difference in HBsAg clearance or seroconversion between PEG-IFNα/LAM combination therapy and PEG-IFNα monotherapy. PEG-IFNα was obviously superior to conventional IFNα in HBsAg clearance, but not in HBsAg seroconversion. Although PEG-IFNα produced significantly higher rates of HBsAg clearance and seroconversion, the absolute change in the proportion of HBsAg clearance and seroconversion was low (about 3-6%). Therefore, additional interventions are needed to improve the rate of positive outcomes.

## Background

Antiviral therapy has been recognized as the first choice for chronic hepatitis B (CHB) treatment and approved as an efficient approach to ameliorating hepatic inflammation and fibrosis, further preventing liver cirrhosis and hepatocellular carcinoma [1-4]. The state of hepatitis B virus (HBV) DNA < 2,000 IU/mL and alanine aminotransferase (ALT) normalization after treatment is a good prediction in both HBeAg-positive and HBeAg-negative CHB [5-9]. Nevertheless, HBsAg clearance and seroconversion, characterized by the loss of serum HBsAg with or without anti-HBs antibody development, are the main markers of a successful immunological response to HBV infection and the closest outcome to clinical cure [10-14]. Some researches indicated that interferon-based therapy, especially pegylated interferon alpha (peginterferon α, PEG-IFNα), obviously outstripped nucleos(t)ide analogues in achieving HBsAg clearance and seroconversion [15-23]. The weekly administration of PEG-IFNα is likely to improve patients' compliance rate, while obtains much better pharmacokinetics [24]. Therefore, several practice guidelines for the management of CHB have considered PEG-IFNα as a first-line therapy for CHB patients [25,26]. However, it remains unclear whether the rate of HBsAg clearance and seroconversion is higher in CHB patients receiving PEG-IFNα-based therapy than in those receiving conventional IFNα or nucleos(t)ide analogues.

In this study, we aimed to elucidate the efficacy of PEG-IFNα-based therapy in the treatment of CHB. Furthermore, our analysis also focused on the rates of HBsAg clearance and seroconversion, which might benefit to optimize the antiviral treatments for CHB. We restricted the comparison groups to PEG-IFNα-based therapy and conventional IFNα or lamivudine (LAM) treatments due to no available controlled clinical trials of PEG-IFNα-based therapy compared with nucleos(t)ide analogues except LAM.

## Methods

### Literature retrieval and study design

Two researchers independently operated the literature retrieval, trial selection and data extraction, reaching to consensus by conferring with each other when discrepancies appeared. The researchers performed a systemic literature retrieval using electronic databases including PubMed (1966-2010), English medical Current Contents (EMCC 1995-2010), China National Knowledge Infrastructure (CNKI 1979-2010), China Hospital Knowledge Database (CHKD 1994-2010) and the Cochrane library clinical trials registry (Issue 3 of 4, Jul 2010). The retrieval was finished in November 2010. The following keywords were used: 'hepatitis B', 'peginterferon', 'pegylated interferon', and 'HBsAg'. In addition, a manual search was conducted using citations in previous publications.

The included studies were divided into different groups according to intervention treatments. The prognosis of the patients were recorded and analysed. Data were extracted by study methodology and defined efficacy measures. Only data regarding the regimens in question were extracted, while data concerning other regimens were also reviewed, and if they were found to be of significance to our study, were noted and discussed. Separate meta-analyses were performed for each group.

### Criteria for inclusion and exclusion

The inclusion criteria were as follows: (i) study design: controlled clinical trials; (ii) study population: CHB patients; (iii) intervention: PEG-IFNα combined with LAM therapy versus PEG-IFNα or LAM monotherapy, PEG-IFNα versus IFNα or LAM monotherapy; (iv) outcomes: HBsAg clearance or seroconversion.

The exclusion criteria were as follows: (i) study design: non-clinical studies; (ii) study population: non-adult population, women with pregnancy or lactation, patients received liver transplantation, patients co-infected with hepatitis C virus, hepatitis D virus or human immunodeficiency virus, patients with a history of alcohol or drug abuse, hepatocellular carcinoma, decompensated liver disease, serious medical or psychiatric illness; (iii) intervention: concurrently using corticosteroid, immunosuppressive agents or Chinese herbal medicine; (iv) outcomes: not reporting any of the efficacy measures of HBsAg clearance or seroconversion as defined by the authors; (v) republished studies, or the full text were not available. Our search was restricted by language; citations in languages other than English or Chinese were not included.

### Efficacy measures and definitions

HBsAg clearance was defined as the disappearance of HBsAg from the serum. HBsAg seroconversion was defined as HBsAg disappearance and anti-HBs antibody appearance.

### Study quality and homogeneity

Methodological quality of the included studies was assessed according to the Jadad quality scale [27], an established composite score evaluating randomization, concealment and reporting of patient withdrawal and dropout rates, with more than or equal to 3 scores defined as high-quality. We performed a sensitivity analysis of quality by considering all studies to be of high-quality. Heterogeneity was assessed for each analysis.

### Statistical analyses

Quantitative meta-analyses were performed to assess differences between PEG-IFNα-based therapy and IFNα or LAM monotherapy groups. Statistical analysis was performed and the Forest plots were generated using the software of Review Manager (RevMan 5.0.24.0, the Nordic Cochrane Center, Rigshospitalet). The odds ratios (OR) were calculated along with their respective 95% confidence intervals (CI) and presented for each individual study. If a clinical trial has no subject (0%) developing the outcome of concern in either of the two comparison groups, we just input the "zero" to the computer, then it will add 0.5 to each of the four cells in the 2 × 2 table by RevMan 5.0.24.0 automatically. Statistical heterogeneity between trials was evaluated by the chi-square (χ^2^) and I square (I^2^) tests, with significance being set at *P *< 0.10. In the absence of statistically significant heterogeneity, the fixed-effect method was used to combine the results. When heterogeneity was confirmed (*P *= 0.10 or lower), the random-effect method was used. Publication bias was assessed by funnel plots.

## Results

### Study selection and characteristics

**Figure 1:**
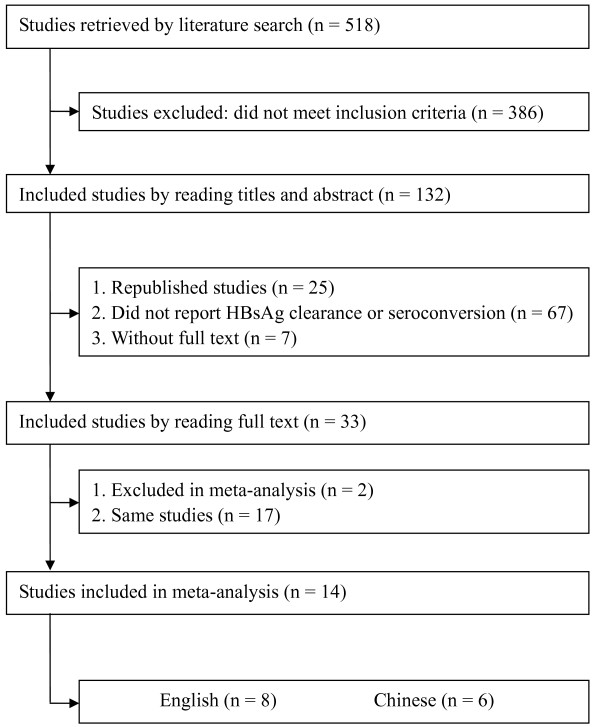
**Literature search and data extraction**.

Our electronic and manual searches identified 518 articles. Thirty-three potentially eligible controlled clinical trials using PEG-IFNα-based therapy for chronic HBV infection were selected, of which nineteen were excluded. Seventeen were duplicate publications, the other two trials were excluded because the interventions contained different adefovir dipivoxil-based therapy and could not be analyzed using meta-analysis for the marked heterogeneity with the included trials. The flow diagram was shown in Figure 1. Therefore, fourteen trials involving a total of 2,682 patients were analyzed in this study. Among these trials, five presented both HBsAg clearance and seroconversion [16,21,22,28,29], eight displayed HBsAg clearance [17-20,23,30-32] and one showed HBsAg seroconversion [15]. Three trials were conducted for long-term follow-up (average 3 years) [17,19,30], the others for 0-72 weeks [15,16,18,20-23,28,29,31,32]. Patients from eleven trials were treated for 48 to 60 weeks [15-20,22,28,30-32], and the rest were treated for 24 weeks [21,23,29]. Of these studies, seven were high-quality and seven were low-quality (Jadad scores of 3-5 and Jadad scores < 3, respectively). All studies were published as full publications, with eight in English [15-19,28,30,31] and six in Chinese [20-23,29,32]. Four studies used sequential therapy [18,19,29,32], and six studies used concurrent therapy [15-17,28,30,31] [Tables 1 and 2].

**Table 1:** Characteristics of studies included in the meta-analysis

Study	n	Study design	Jadad score	Therapy period	Follow-up period	Therapy regimen
Lau G.K.K. 2005	814	RCT	4	48 w	24 w	1. PEG-IFNα-2a (180 ug/w) + placebo 2. PEG-IFNα-2a (180 ug/w) + LAM (100 mg/d) 3. LAM (100 mg/d)
Marcellin P. 2004	537	RCT	4	48 w	24 w	1. PEG-IFNα-2a (180 ug/w) + placebo 2. PEG-IFNα-2a (180 ug/w) + LAM (100 mg/d) 3. LAM (100 mg/d)
Marcellin P. 2009	315	RCT	4	48 w	3 y	1. PEG-IFNα-2a (180 ug/w) + placebo 2. PEG-IFNα-2a (180 ug/w) + LAM (100 mg/d) 3. LAM (100 mg/d)
Chan H.L.Y.^1 ^2005	100	RCT	3	52-60 w	24 w	1. PEG-IFNα-2b (1.5 ug/kg, 1/w, Wt < 65 kg; 100 ug/w, Wt ≥ 65 kg) 8 w → PEG-IFNα-2b + LAM 24 w → LAM 28 w 2. LAM (100 mg/d)
Chan H.L.Y.^2 ^2005	95	RCT	3	52-60 w	3 y	1. PEG-IFNα-2b (1.5 ug/kg, 1/w, Wt < 65 kg; 100 ug/w, Wt ≥ 65 kg) 8 w → PEG-IFNα-2b + LAM 24 w → LAM 28 w 2. LAM (100 mg/d)
Janssen HLA 2005	266	RCT	5	52 w	26 w	1. PEG-IFNα-2b (100 ug/w) + placebo 2. PEG-IFNα-2b (100 ug/w) + LAM (100 mg/d)
Buster E.H.C.J 2008	172	RCT	5	52 w	3.0 ± 0.8 y	1. PEG-IFNα-2b (100 ug/w) + placebo 2. PEG-IFNα-2b (100 ug/w) + LAM (100 mg/d)
Kaymakoglu S. 2007	48	RCT	2	48 w	24 w	1. PEG-IFNα-2b (1.5 ug/kg, 1/w) 2. PEG-IFNα-2b (1.5 ug/kg, 1/w) + LAM (100 mg/d)
Tian YL 2007	72	RCT	2	48 w	72 w	1. PEG-IFNα-2a (180 ug/w) 2. Conventional IFNα-2a (5 MU, 1/2 d)
Huang ZL 2010	50	NRCT	1	52 w	0	1. PEG-IFNα-2a (135 ug/w) 12 w → PEG-IFNα-2a + LAM (100 mg/d) 12 w → PEG-IFNα-2a 28 w 2. PEG-IFNα-2a 52 w
Cui JJ 2006	80	RCT	2	6 m	6 m	1. PEG-IFNα-2a (180 ug/w) 2. Conventional IFNα-2a (5 MU, 3/w)
Li ZQ 2010	74	NRCT	1	48 w	24 w	1. PEG-IFNα-2a (180 ug/w) 2. Conventional IFNα-2a (5 MU, 1/2 d)
Guan LJ 2006	32	RCT	2	24 w	6 m	1. PEG-IFNα-2a (180 ug/w) 2. Conventional IFNα-2b (5 MU, 1/2 d)
Shi XF 2006	27	NRCT	1	6 m	0	1. PEG-IFNα-2a (135-180 ug/w) + placebo 2. PEG-IFNα-2a (135-180 ug/w) + LAM (100 mg/d) 3 m → PEG-IFNα-2a 3 m

PEG-IFNα-2a: pegylated interferon alpha-2a; LAM: lamivudine; RCT: randomized controlled trial; NRCT: nonrandomized controlled trial; d: days; w: weeks; m: months; y: years.

**Table 2:** Patient selection criteria of studies included in the meta-analysis

Study	Inclusion criteria	Exclusion criteria
Lau G.K.K. 2005	1. Adults	1. Treatment within 6 m
	2. HBsAg positive for > 6 m and HBeAg positive	2. HIV infection, hepatitis C or D
	3. HBV DNA > 500 000 copies/ml	3. Decompensated liver disease
	4. Evidence of inflammation on biopsy and 1 ULN < ALT < 10 ULN	4. Serious medical or psychiatric illness
		5. Alcohol or drug use within 1 y
		6. Neutrophils < 1500/mm^3^, platelets < 90 000/mm^3^, or creatinine > 1.5 ULN
Marcellin P. 2004	1. Adults	1. Treatment within 6 m
	2. HBsAg positive for > 6 m and HBeAg negative	2. HIV infection, hepatitis C or D
	3. HBV DNA > 100 000 copies/ml	3. Decompensated liver disease
	4. Evidence of inflammation on biopsy and 1 UNL < ALT < 10 ULN	4. Serious medical or psychiatric illness
		5. Alcohol or drug use within 1 y
		6. Neutrophils < 1500/mm^3^, platelets < 90 000/mm^3^, or creatinine > 1.5 ULN
Marcellin P. 2009	1. Adults	1. Treatment within 6 m
	2. HBsAg positive for > 6 m and HBeAg negative	2. HIV infection, hepatitis C or D
	3. HBV DNA > 100 000 copies/ml	3. Decompensated liver disease
	4. Evidence of inflammation on biopsy and 1 ULN < ALT < 10 ULN	4. Serious medical or psychiatric illness
		5. Alcohol or drug use within 1 y
		6. Neutrophils < 1500/mm^3^, platelets < 90 000/mm^3^, or creatinine > 1.5 ULN
Chan H.L.Y. ^1 ^2005	1. 18-65 years old	1. Decompensated liver disease or a history of interferon or antiviral agent use
	2. HBsAg positive for > 6 m and HBeAg positive	2. HIV infection, hepatitis C or D
	3. HBV DNA > 500 000 copies/ml	3. History of hepatocellular carcinoma
	4. 1.3 ULN < ALT < 5 ULN	4. Other causes of liver disease
		5. Serious medical or psychiatric illness
		6. Concurrent use of corticosteroid or immunosuppressive agents
		7. Pregnancy
Chan H.L.Y.^2 ^2005	1. 18-65 years old	1. Decompensated liver disease or a history of interferon or antiviral agent use
	2. HBsAg positive for > 6 m and HBeAg positive	2. HIV infection, hepatitis C or D
	3. HBV DNA > 500 000 copies/ml	3. History of hepatocellular carcinoma
	4. 1.3 ULN < ALT < 5 ULN	4. Other causes of liver disease
		5. Serious medical or psychiatric illness
		6. Concurrent use of corticosteroid or immunosuppressive agents
		7. Pregnancy
Janssen HLA 2005	1. ≥ 16 years old	1. Antiviral or immunosuppressive therapy within 6 m
	2. HBsAg positive for > 6 m and HBeAg positive on two occasions within 8 w of randomization	2. HIV infection, hepatitis C or D
	3. Evidence of inflammation by two measurements of ALT > 2 ULN within 8 w of randomization	3. Advanced liver disease or carcinoma
		4. Serious medical or psychiatric illness, or uncontrolled thyroid disease
		5. Substance abuse within 2 y
		6. Pregnancy or inadequate contraception
		7. Leucocytes ≤ 3000/mm^3^, neutrophils ≤ 1800/mm^3^, or platelets ≤ 100 000/mm^3^
Buster E.H.C.J 2008	1. ≥ 16 years old	1. Antiviral or immunosuppressive therapy within 6 m
	2. HBsAg positive for > 6 m and HBeAg positive on two occasions within 8 w of randomization	2. HIV infection, hepatitis C or D
	3. Evidence of inflammation by two measurements of ALT > 2 ULN within 8 w of randomization	3. Advanced liver disease or carcinoma
		4. Serious medical or psychiatric illness, or uncontrolled thyroid disease
		5. Substance abuse within 2 y
		6. Pregnancy or inadequate contraception
		7. Leucocytes ≤ 3000/mm^3^, neutrophils ≤ 1800/mm^3^, or platelets ≤ 100 000/mm^3^
Kaymakoglu S. 2007	1. ≥ 18 years old	1. Other cause of chronic liver disease
	2. HBsAg positive for > 6 m, HBeAg negativity on two occasions in the past 3 m, ALT > 1.3 ULN on two occasions during the preceding 3 m	2. Received immunosuppressive or antiviral treatment in the previous 6 m
	3. HBV DNA positivity (lower limit of detection [LLD], 4 pg/ml)	3. Exhibited hepatocellular carcinoma
	4. Compensated liver disease with histological evidence of chronic hepatitis	
Tian YL 2007	1. 18-60 years old	1. Pregnancy or lactation
	2. Without using antiviral or immunosuppressive therapy within 6 m	2. Serum albumin < 3.5 g/L; PT prolong ≥ 4 s; serum bilirubin > 34 μmol/L; pretreatment neutrophils < 1.5 × 10^9^/L; Platelets < 90 × 10^9^/L; hemoglobin (male) < 125 g/L, (female) < 115 g/L
	3. HBV DNA > 500 000 copies/ml	3. Hepatitis A, C, D or E
	4. At least two occasions of ALT > 100-500 U/L within 6 m before treatment, and at least one occasion within 35 d before treatment, interval time ≥ 14 d	4. Serious medical or psychiatric illness
		5. Autoimmune liver disease or alcoholic hepatitis
Huang ZL 2010	1. HBsAg positive for > 6 m and HBeAg positive	1. Leucocytes ≤ 3.0 × 10^9^/L, platelets ≤ 75 × 10^9^/L
	2. HBV DNA > 100 000 copies/ml	2. Pregnancy or lactation
	3. Without using antiviral therapy within 6 m and ALT > 2 ULN within 3 m	3. HIV infection, other viral hepatitis
		4. Malignant tumor
		5. Serious medical or psychiatric illness, or uncontrolled thyroid disease, diabetes mellitus, autoimmune disease et al
Cui JJ 2006	1. HBsAg positive for > 6 m, HBeAg positive, HBV DNA > 100 000 copies/ml	Not reported
	2. 2 ULN < ALT < 10 ULN	
	3. Without other viral hepatitis or HIV infection	
	4. Without using antiviral or immunosuppressive therapy within 6 m	
	5. Neutrophils > 15 × 10^9^/L, Platelets > 90 × 10^9^/L	
	6. Without serious medical, psychiatric illness or decompensated liver disease	
Li ZQ 2010	1. HBsAg positive for > 6 m, HBeAg positive	Not reported
	2. ALT > 80 U/L, HBV DNA > 10^4^IU/ml	
	3. Without other viral hepatitis	
	4. Without using antiviral or immunosuppressive therapy within 6 m	
Guan LJ 2006	1. Course of disease is 2-8 y	Not reported
	2. 18-55 years old	
	3. Pretreatment 1.5 ULN < ALT < 10 ULN, HBsAg positive, HBeAg negative, HBV DNA > 100 000 copies/ml	
	4. Without other viral hepatitis	
	5. Without using antiviral therapy pretreatment and without contraindication of antiviral therapy	
Shi XF 2006	1. 16-60 years old	1. Decompensated liver disease
	2. Without using antiviral therapy within 6 m or virological relapse or YMDD mutation after more than 1 y LAM treatment	2. Serious medical or psychiatric illness
	3. HBsAg positive, HBeAg positive	3. Neutrophils < 1500/mm^3^, Platelets ≤ 70 000/mm^3^
	4. HBV DNA > 100 000 copies/ml, or relapse (increase 2 log 10 copies/ml)	4. Hepatitis A, C, D or E
	5. Serum total bilirubin < 40 umol/L	5. alcohol or drugs abuse within 1 y
	6. PEG-IFNα-2a monotherapy group 2 ULN < ALT < 10 ULN; PEG-IFNα-2a combination with LAM group ALT < 10 ULN	

HBsAg: hepatitis B surface antigen; HBeAg: hepatitis B e antigen; HBV: hepatitis B virus; ALT: alanine aminotransferase; ULN: upper limit of normal; HIV: human immunodeficiency virus; PEG-IFNα-2a: pegylated interferon alpha-2a; LAM: lamivudine; d: days; m: months; y: years.

### HBsAg clearance and seroconversion in patients receiving combination therapy or monotherapies

#### PEG-IFNα+LAM combination therapy vs. PEG-IFNα monotherapy

**Figure 2:**
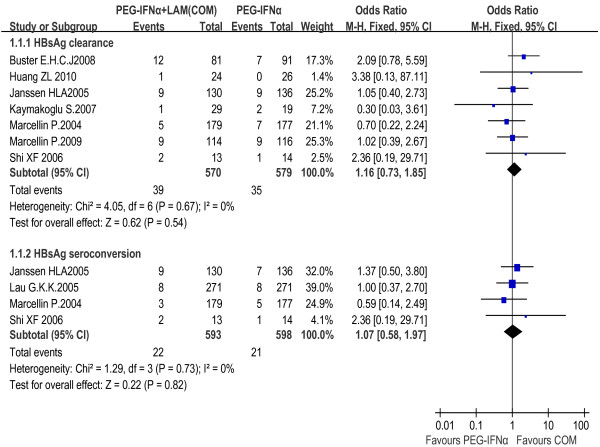
**Analysis of HBsAg clearance and HBsAg seroconversion between PEG-IFNα+LAM combination therapy and PEG-IFNα monotherapy (overall trials)**.

**Figure 3:**
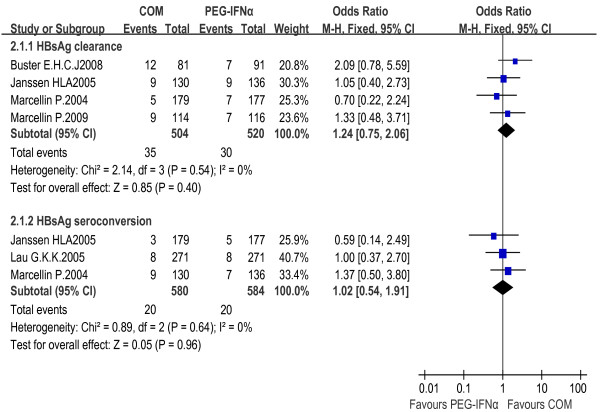
**Analysis of HBsAg clearance and HBsAg seroconversion between PEG-IFNα+LAM combination therapy and PEG-IFNα monotherapy (high-quality studies)**.

The rates of HBsAg clearance and seroconversion were 6.8% and 3.7% in the combination therapy group, and 6.0% and 3.5% in the PEG-IFNα monotherapy group, respectively. The fixed effect model for meta-analysis was used according to the heterogeneity test (χ^2 ^= 4.05, df = 6, *P *= 0.67, I^2 ^= 0% and χ^2 ^= 1.29, df = 3, *P *= 0.73, I^2 ^= 0%, respectively). The difference between the two groups did not show statistical significance neither in HBsAg clearance rate [OR = 1.16, 95% CI (0.73-1.85), *P *= 0.54] nor in seroconversion rate [OR = 1.07, 95% CI (0.58-1.97), *P *= 0.82] [Figure 2]. We also conducted a sensitivity analysis including high-quality studies only and obtained the same result as the overall trials [Figure 3].

#### PEG-IFNα+LAM combination therapy vs. LAM monotherapy

In comparison with the LAM monotherapy, the combination therapy led to higher HBsAg clearance rates during follow-up (2.6% vs. 0% at 24 weeks, and 6.2% vs. 0% at 3 years, respectively). Thus, PEG-IFNα combined with LAM therapy was significantly more effective than LAM monotherapy in HBsAg eradication, especially in long-term follow-ups [OR = 7.24, 95% CI (0.88-59.28), *P *= 0.07, and OR = 9.41, 95% CI (1.18-74.94), *P *= 0.03, respectively]. The fixed effect model for meta-analysis was used according to the heterogeneity test (χ^2 ^= 0.37, df = 1, *P *= 0.54, I^2 ^= 0% and χ^2 ^= 0.60, df = 1, *P *= 0.44, I^2 ^= 0%, respectively).

**Figure 4:**
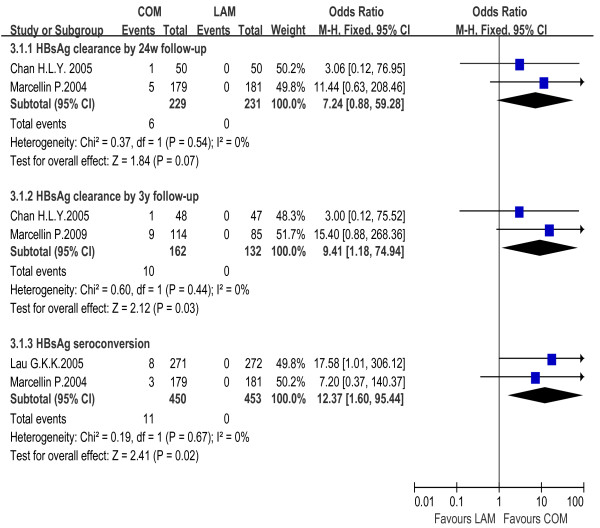
**Analysis of HBsAg clearance and HBsAg seroconversion between PEG-IFNα+LAM combination therapy and LAM monotherapy**.

Similarly, HBsAg seroconversion rate was 2.4% in the combination therapy group and 0% in the LAM monotherapy group. Heterogeneities were assessed and no concern was found (χ^2 ^= 0.19, df = 1, *P *= 0.67, I^2 ^= 0%), thus allowing the use of a fixed effect model for meta-analysis. The difference reached statistical significance [OR = 12.37, 95% CI (1.60-95.44), *P *= 0.02] [Figure 4].

#### PEG-IFNα monotherapy vs. IFNα monotherapy

**Figure 5:**
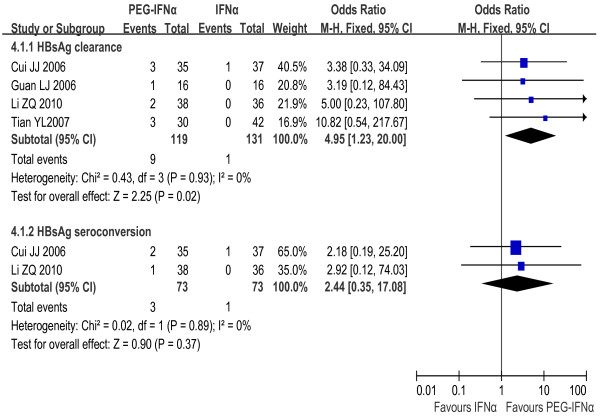
**Analysis of HBsAg clearance and HBsAg seroconversion between PEG-IFNα and IFNα monotherapy**.

The rates of both HBsAg clearance and HBsAg seroconversion in the PEG-IFNα monotherapy group (7.6% and 4.1%) exceeded the IFNα monotherapy group (0.8% and 1.4%). The fixed effect model for meta-analysis was used according to the heterogeneity test (χ^2 ^= 0.43, df = 3, *P *= 0.93, I^2 ^= 0% and χ^2 ^= 0.02, df = 1, *P *= 0.89, I^2 ^= 0%, respectively). Significant difference in HBsAg clearance rates was observed between the two groups [OR = 4.95, 95% CI (1.23-20.00), *P *= 0.02], but not in seroconversion rates [OR = 2.44, 95% CI (0.35-17.08), *P *= 0.37] [Figure 5].

#### PEG-IFNα monotherapy vs. LAM monotherapy

**Figure 6:**
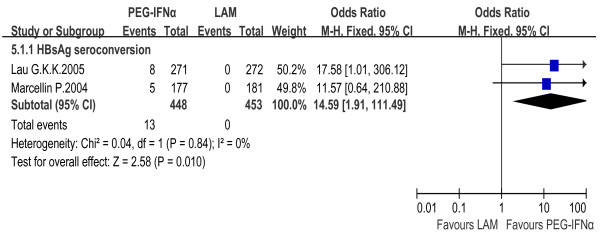
**Analysis of HBsAg seroconversion between PEG-IFNα and LAM monotherapy**.

HBsAg seroconversion rate was 2.9% in PEG-IFNα monotherapy group and 0% in LAM monotherapy group. The fixed effect model for meta-analysis was used according to the heterogeneity test (χ^2 ^= 0.04, df = 1, *P *= 0.84, I^2 ^= 0%). The difference between the two groups reached statistical significance [OR = 14.59, 95% CI (1.91-111.49), *P *= 0.01] [Figure 6].

### Publication bias

**Figure 7:**
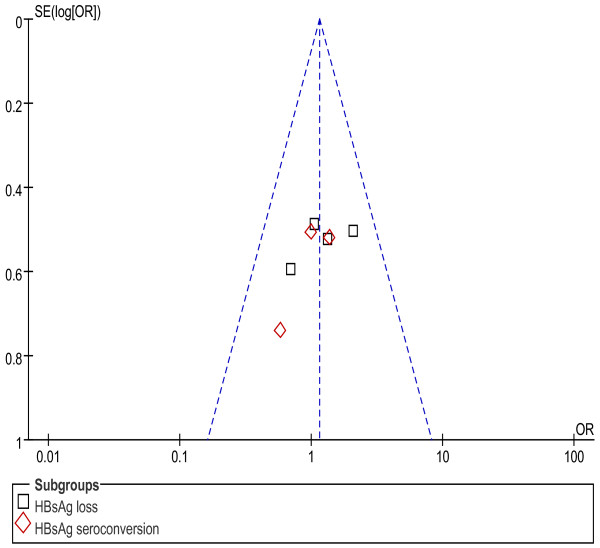
**Assessment of publication bias for PEG-IFNα+LAM combination therapy versus PEG-IFNα monotherapy on HBsAg clearance and HBsAg seroconversion (high-quality studies)**.

An assessment of publication bias was conducted using funnel plots. Evidences of publication bias based on the funnel plots were found in comparison of combination therapy with PEG-IFNα monotherapy on HBsAg clearance and HBsAg seroconversion only among high-quality studies [Figure 7]. There was no apparent publication bias in the other comparison groups.

## Discussion

Currently there are two kinds of antiviral agents for the treatment of CHB patients, interferon (conventional or pegylated interferon) and nucleos(t)ide analogues (lamivudine, telbivudine, adefovir, entecavir, and tenofovir). It has been documented that interferon is more efficient than nucleos(t)ide analogues on HBeAg clearance and HBeAg seroconversion. However, the use of interferon was restricted because of the inconvenience of injection and risk of adverse reactions. Nucleos(t)ide analogues were more convenient to use and strongly suppressed HBV DNA replication. However, nucleos(t)ide analogues had the disadvantages of developing resistance and there is no established course of treatment. Combination therapy with interferon and nucleos(t)ide analogues was prompted in order to enhance the therapeutic efficacy.

HBsAg clearance or seroconversion, which might predict intrahepatic covalently closed circular DNA (cccDNA) reduction, is the ultimate outcome of antiviral therapy. However, there are not sufficient studies involved in this objective. Compared with any single study, meta-analysis has increased power for statistical tests, and increased precision for confidence intervals, because the conclusions often reflect a broad spectrum of patients and study characteristics, and the results are more generalizable than a single study. Our study is the first to analyse PEG-IFNα-based therapy for CHB treatment focusing on HBsAg clearance or seroconversion, pooling data from all pertinent clinical-controlled trials into meta-analysis. This analysis is to achieve evidence-based conclusions on the matter, to resolve the controversy over the selection of antiviral regimens, and to be further referred to by future clinical investigations.

In this analysis, we found that PEG-IFNα monotherapy and combination with PEG-IFNα and LAM obtained comparable efficacies, achieving similar rates of HBsAg clearance and seroconversion (*P *= 0.54 and 0.82 respectively) [15-17,28-32]. We also set up subgroups for meta-analysis according to follow-up terms, but no significant difference was obtained. Thus, the addition of LAM to PEG-IFNα regimen could not promote the clinical cure in defined course of treatment but brought heavy financial burden to patients. Up to date, there was seldom reports of randomized controlled clinical trials based on combination therapy with PEG-IFNα and other nucleos(t)ide analogues. Paola Piccolo et al [33] performed a multicenter randomized controlled trial, in which a total of 60 patients were included and efficacies of PEG-IFNα monotherapy and combination therapy with PEG-IFNα and adefovir dipivoxil were compared. Only one patient (3.3%) in the combination therapy group obtained HBsAg clearance during 24 w follow-up. The combination of PEG-IFNα with more potential nucleos(t)ide analogues such as entecavir or tenofovir needs to be further investigated in large, randomized studies. Nucleos(t)ide analogues typically demand to be continued indefinitely to maintain viral suppression, without a clearly defined endpoint for stopping treatment. This approach increases the risk of antiviral resistance, and the safety of long-term therapy with nucleos(t)ide analogues also needs to be explored.

Higher rates of HBsAg clearance and seroconversion were observed in patients receiving the combination regimen as compared with those receiving LAM monotherapy [15-19]. Increasing HBsAg loss rate was only obtained by long-term follow-up [17,19]. Patients treated with PEG-IFNα monotherapy achieved higher rate of HBsAg seroconversion as compared with those treated with LAM monotherapy [15,16]. The difference in HBsAg clearance or seroconversion rates might relate to antiviral mechanisms of the agents. There are two mechanisms of interferon action: (i) direct antiviral effect by suppressing synthesis of viral DNA and by activating antiviral enzymes; and (ii) magnification of the cellular immune response against hepatocytes infected with HBV [34]. PEG-IFNα-based therapy exerts persistently an immunomodulatory activity to produce antibodies to HBV antigens and sustained virological response. Otherwise, nucleos(t)ide analogues, such as LAM, suppresses HBV replication by inhibiting the reverse transcriptase activity of HBV polymerase. Only a few patients sustained the response and HBsAg loss after 6 months of treatment discontinuation [35,36]. The same conclusions were drawn from comparisons between PEG-IFNα and adefovir dipivoxil or entecavir [37-39]. Complementary activity of immunoregulation and sustained suppression of HBV replication in CHB patients can be achieved by combination of PEG-IFNα and nucleos(t)ide analogues, which can initiate specific immune response to bring about HBsAg elimination and seroconversion.

Clearance rate of HBsAg was significantly higher in patients receiving PEG-IFNα monotherapy than in those receiving conventional IFNα monotherapy [20-23], which might attribute to their different pharmacokinetics. Conventional IFNα was administered once every other day, and it reached peak concentration rapidly in the blood and got lower at intervals. PEG-IFNα was used once per week, with its effective blood concentration persisting for a week [24]. Currently, the updated therapeutic guidelines on management of CHB suggest that PEG-IFNα should be administered instead of conventional IFNα to improve antiviral response.

Three studies providing 3-year follow-up data were included for the analysis of HBsAg clearance rate in our study. Higher rate of HBsAg clearance was observed in patients with the combination therapy as compared with those using LAM monotherapy (*P *= 0.03), but no significant difference was observed after short-term follow-up investigation (*P *= 0.07). The sustained long-term efficacy of PEG-IFNα-based therapy was reflected by increased proportion of patients achieving HBsAg clearance during follow-up [17,30,40,41]. It may attribute to the effect of PEG-IFNα since HBsAg clearance was rarely obtained in patients treated with lamivudine, adefovir, entecavir or tenofovir [42-44]. It also indicated that long-term follow-up is necessary to achieve evidence of optimized antiviral strategy.

Our study contains several limitations. Firstly, although no difference was found between the high-quality studies and the overall trials in the comparison of the combination therapy group and PEG-IFNα monotherapy group, the low-quality studies in our analysis, especially those in Chinese publications, which lacked randomization, may weaken our conclusions. Secondly, while this study focused on PEG-IFNα and LAM combination therapy, combinations of PEG-IFNα with other potent antiviral agents need to be further explored, which was limited by deficient data currently available. Finally, the absence of adequate controlled trials precluded our analysis on the subsets of PEG-IFNα-2a or 2b, as well as HBeAg positive or negative CHB patients.

## Conclusions

PEG-IFNα facilitated HBsAg clearance or seroconversion in CHB patients. PEG-IFNα-based therapy was more effective than LAM monotherapy in achieving HBsAg clearance or seroconversion for both HBeAg-positive and HBeAg-negative CHB patients. There was no significant difference in HBsAg clearance or seroconversion between the combination therapy and PEG-IFNα monotherapy. PEG-IFNα was obviously superior to conventional IFNα in HBsAg clearance, but not in HBsAg seroconversion. Although PEG-IFNα produced significantly higher rates of HBsAg clearance and seroconversion, the absolute change in the proportion of HBsAg clearance and seroconversion was low (about 3-6%). Therefore, additional interventions are needed to improve positive outcomes.

## List of Abbreviations

IFNα: interferon alpha; PEG-IFNα: pegylated interferon alpha, peginterferon α; LAM: lamivudine; CHB: chronic hepatitis B; HBsAg: hepatitis B surface antigen; HBeAg: hepatitis B e antigen; HBV: hepatitis B virus; cccDNA: covalently closed circular DNA; ALT: alanine aminotransferase; ULN: upper limit of normal; HIV: human immunodeficiency virus; OR: odds ratio; CI: confidence intervals; χ^2^: chi-square; I^2^: I square; RCT: randomized controlled trial; NRCT: nonrandomized controlled trial; d: days; w: weeks; m: months; y: years.

## Competing interests

The authors report no conflicts of interest. The authors alone are responsible for the content and writing of the paper.

## Authors' contributions

YN designed the research; WL and WR operated the literature retrieval, trial selection and data extraction; WL, MW, LK and YZ analyzed data; WL, MW, LK, WR and YZ wrote the paper. All authors read and approved the final manuscript.

## Pre-publication history

The pre-publication history for this paper can be accessed here:

http://www.biomedcentral.com/1471-2334/11/165/prepub
